# BMI1 enables interspecies chimerism with human pluripotent stem cells

**DOI:** 10.1038/s41467-018-07098-w

**Published:** 2018-11-07

**Authors:** Ke Huang, Yanling Zhu, Yanlin Ma, Bentian Zhao, Nana Fan, Yuhang Li, Hong Song, Shilong Chu, Zhen Ouyang, Quanjun Zhang, Qi Xing, Chengdan Lai, Nan Li, Tian Zhang, Jiaming Gu, Baoqiang Kang, Yongli Shan, Keyu Lai, Wenhao Huang, Yuchan Mai, Qing Wang, Jinbing Li, Aiping Lin, Yanqi Zhang, Xiaofen Zhong, Baojian Liao, Liangxue Lai, Jiekai Chen, Duanqing Pei, Guangjin Pan

**Affiliations:** 1 Key Laboratory of Regenerative Biology of Chinese Academy of Sciences, Joint School of Life Sciences, Guangzhou Institutes of Biomedicine and Health, Chinese Academy of Sciences, Guangzhou Medical University, 510530 Guangzhou, China; 2 Guangdong Provincial Key Laboratory of Stem Cell and Regenerative Medicine, South China Institute for Stem Cell Biology and Regenerative Medicine, Guangzhou Institutes of Biomedicine and Health, Chinese Academy of Sciences, 510530 Guangzhou, China; 3 Institute for Stem Cell and Regeneration, Chinese Academy of Sciences, 100101 Beijing, China; 4 Hefei Institute of Stem Cell and Regenerative Medicine, Guangzhou Institutes of Biomedicine and Health, Chinese Academy of Sciences, 510530 Guangzhou, China; 5 University of Chinese Academy of Sciences, 100049 Beijing, China; 6 Hainan Provincial Key Laboratory for Human Reproductive Medicine and Genetic Research, The First Affiliated Hospital of Hainan Medical University, Hainan Medical University, 570102 Haikou, Hainan China

## Abstract

Human pluripotent stem cells (hPSCs) exhibit very limited contribution to interspecies chimeras. One explanation is that the conventional hPSCs are in a primed state and so unable  to form chimeras in pre-implantation embryos. Here, we show that the conventional hPSCs undergo rapid apoptosis when injected into mouse pre-implantation embryos. While, forced-expression of BMI1, a polycomb factor in hPSCs overcomes the apoptosis and enables hPSCs to integrate into mouse pre-implantation embryos and subsequently contribute to chimeras with both embryonic and extra-embryonic tissues. In addition, BMI1 also enables hPSCs to integrate into pre-implantation embryos of other species, such as rabbit and pig. Notably, BMI1 high expression and anti-apoptosis are also indicators for naïve hPSCs to form chimera in mouse embryos. Together, our findings reveal that the apoptosis is an initial barrier in interspecies chimerism using hPSCs and provide a rational to improve it.

## Abstract (web summary)

Conventional human pluripotent stem cells (hPSCs) fail to contribute to interspecies chimaeras when injected into mouse blastocysts. Here the authors show that forced expression of BMI1 overcomes apoptosis of hPSCs in blastocysts of mouse, rabbit and pig allowing them to contribute to chimaeras.

## Introduction

Generation of embryonic chimeras provides an approach with both conceptual and practical importance to fully assess the developmental potential of the introduced cells^1–4^. More importantly, interspecies chimeras using human pluripotent stem cells (hPSCs) hold the potential to generate humanized organs for regenerative medicine by blastocyst complementation^4,5^. It is well known that a successful chimera formation largely relies on the state of the introduced PSCs. Currently, most PSCs cultured in vitro are known to represent two major different states of pluripotency. For example, mouse ESCs, deriving from preimplantation blastocysts are considered to be in a naïve state while epiblast stem cells (EpiSCs) from postimplantation egg cylinders are in a primed state^6^. Naïve and primed PSCs harbor distinct development potential in chimera assays. Naïve mESCs can integrate into the early blastocysts and contribute to all embryonic tissues during subsequent development. In contrast, the primed EpiSCs fail to integrate into the preimplantation blastocysts, but could integrate well into the postimplantation embryos^7,8^. Therefore, it is presumed that matching of the developmental stage is critical in chimera formation, i.e., the PSCs need to be introduced into the embryos with the particular stage from where they were derived^4^. Indeed, the mouse EpiSCs underwent apoptosis when injected into an unmatched preimplantation blastocyst^9^ and inhibition of the apoptosis enabled mouse EpiSCs to integrate into the preimplantation blastocyst and form chimeras^10^. In contrast, the conventional hPSCs either induced pluripotent stem cells (iPSCs) or hESCs, even though derived from preimplantation blastocysts, fail to integrate into the same stage of mouse blastocysts^9,11,12^. It is evident that these conventional hPSCs resemble much more to the primed mouse EpiSCs in term of their cultural requirements and gene expression programs^6,13^. Therefore, it might be incompatible to directly inject hPSCs into preimplantation blastocysts for chimera formation. Consistently, hPSCs integrate well into the postimplantation mouse embryos that were cultured in vitro^14^. To date, significant efforts have been made and a number of reports published describing the generation of naïve hPSCs^15–22^. However, despite their gene expression programs, as well as culture requirements and morphology etc. are much closer to that of naïve mESCs, the naïve-like hPSCs still exhibit very poor integration upon injection into mouse blastocysts^9,15,23^. Thus, the major barriers underlying interspecies chimerism using human PSCs remain to be fully illuminated. In this study, we show that the survival rather than the naïve state is the initial barrier in interspecies chimerism using hPSCs. Overcoming apoptosis by BMI1 enables conventional hPSCs to survive and integrate well into the blastocysts of different species, including mouse, rabbit, and pig. In addition, BMI1 expression and antiapoptosis ability are also indicators for those naïve hPSCs that are able to form chimera in mouse embryos.

## Results

### BMI1 enables chimera formation with the conventional hPSCs

**Fig. 1:**
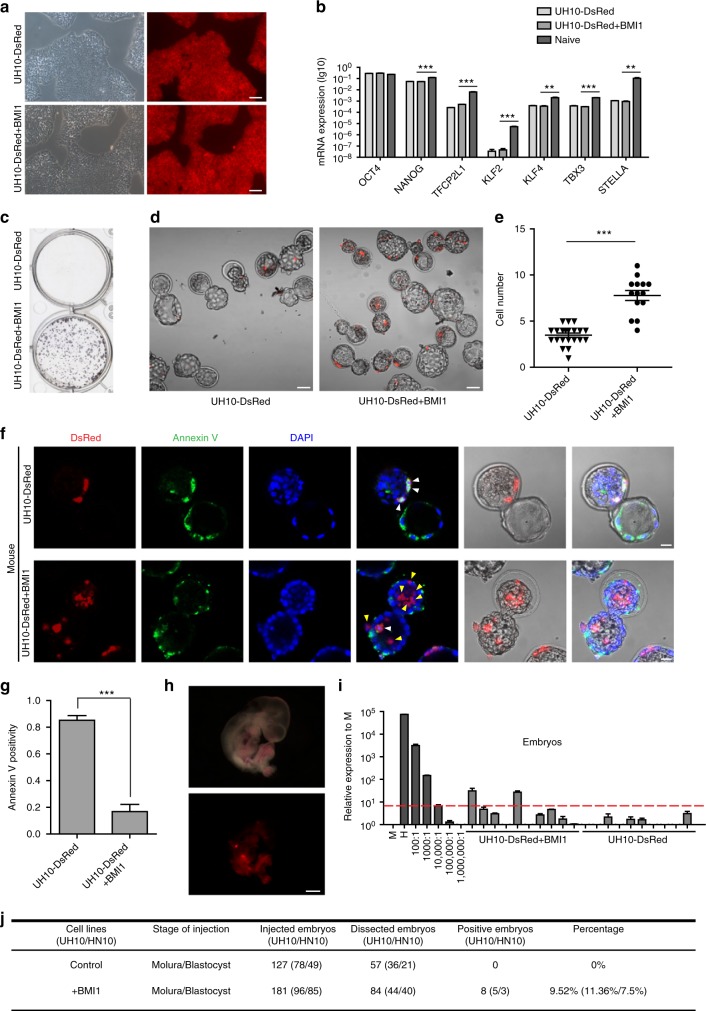
BMI1 inhibits apoptosis and enables chimera formation with hPSCs. **a** Morphology and DsRed fluorescence expression of UH10-DsRed and UH10-DsRed + BMI1 cell lines. Scale bars, 100 µm. **b** RT-qPCR analysis of the selected naïve-state pluripotency markers in the indicated cells. Naïve-state hPSCs were converted according to the protocol from Austin Smith’s lab (3i, Methods). Error bars represent mean + SEM of three independent replicates. **p < 0.01, ***p < 0.001. **c** Alkaline phosphatase staining on colonies formed by the indicated cells after plating at 2000 single cells per well of 6-well-plate for 7 days. **d**, **e** Merged images and statistics of the numbers of DsRed^+^ cells in injected mouse blastocysts after injection of the ten indicated DsRed^+^ cells in the later morulas or early blastocysts and 1-day culture in vitro; scale bars, 50 µm, mean ± SEM of 21 (UH10-DsRed) or 14 (UH10-DsRed + BMI1) samples, ***p < 0.001. **f** Representative fluorescence images of mouse embryos stained with Annexin V after injection of ten indicated DsRed^+^ cells in the later morulas or early blastocysts and 1-day culture in vitro. White arrow, DsRed^+^/Annexin V^+^ cells; Yellow arrow, DsRed^+^/Annexin V^−^ cells. Scale bars, 20 µm. **g** Statistics of the percentage of engrafted DsRed^+^ cells with Annexin V^+^ in the mouse blastocysts; mean + SEM of 21 (UH10-DsRed) or 14 (UH10-DsRed + BMI1) samples, ***p < 0.001. **h** E10.5 mouse embryos derived from UH10-DsRed + BMI1 in bright field and fluorescence. Scale bars, 1 mm. **i** Representative quantitative genomic PCR analysis of the human mitochondria DNA in E10.5 mouse embryos after injection of the indicated cells. A human DNA control (H), a mouse DNA control (M) and a series of human−mouse cell dilutions (1/100 to 1/1000,000) were run in parallel to estimate the degree of human cell integration. The dashed line indicates the detection level of one human cell in 10,000 mouse cells. **j** Summary of chimera assays of the indicated DsRed^+^ cells injected at the later morula or early blastocyst stage. Dox were added in culture or fed to recipient for BMI1 overexpression until analysis, otherwise indicated

It has been reported that apoptosis is one barrier in chimera formation when cells were injected into stage unmatched embryos^10^. We have interests to examine whether it also occurs in hPSC-based interspecies chimerism. We then prepared UH10 hiPSCs that were previously generated in our lab with constitutive expression of a reporter gene, DsRed in AAVS1 locus through gene targeting (UH10-DsRed) (Methods)^24,25^. We have previously shown that BMI1, a polycomb factor could significantly suppress apoptosis triggered by individualization in hESCs^26^. We thus prepared additional hiPSC-DsRed cell line integrated with an inducible BMI1 expression cassette (UH10-DsRed + BMI1) to examine their chimera competence. Both UH10 and UH10-DsRed + BMI1 showed typical morphologies of the conventional hPSCs as well as teratoma formation capability and normal karyotype, but no significant upregulation of known naïve pluripotent specific markers (Fig. 1a, b, Supplementary Fig. 1a−e). Consistent with our previous findings, BMI1 expression dramatically enhanced the survival and cloning efficiency of hiPSCs when plated in single-cell density^26^ (Fig. 1c). We then examined their survival and apoptosis after injection into preimplantation mouse embryos, including later morulas and early blastocysts. After 1-day in vitro culture, UH10-DsRed + BMI1 showed much higher number of viable cells in mouse embryos than the parental UH10-DsRed cells (Fig. 1d, e). Consistently, around 80% of UH10-DsRed cells injected in the mouse later morulas or early blastocysts underwent apoptosis as examined by Annexin V staining (Fig. 1f, g). In contrast, Annexin V-positive cells were significantly reduced in BMI1-expressed hiPSCs (Fig. 1f, g). These data demonstrate that BMI1 overcomes apoptosis and enables the conventional hiPSCs to integrate into mouse preimplantation embryos.

Because BMI1-expressed hPSCs showed normal differentiation^26^, we then examined long-term chimera contribution of UH10-DsRed + BMI1 cells in mouse postimplantation conceptuses. The DsRed-positive cells could be easily observed in E10.5 fetus injected with UH10-DsRed + BMI1 cells (Fig. 1h). The presence of human cells in mouse fetus was further confirmed by the more sensitive mitochondrial PCR assay^23^. In total, human cells can be detected in 11.36% of recovered mouse embryos injected with UH10-DsRed + BMI1 cells using 1/10^4^ as a threshold (Fig. 1i, j, Supplementary Fig. 1f). With the same threshold, no human cells could be detected from all recovered embryos injected with the parental UH10-DsRed cells (Fig. 1i, j, Supplementary Fig. 1f). To confirm these findings, we also examined the role of BMI1 in an additional hPSC cell line, HN10 hESCs^27^. We thus prepared similar BMI1 or DsRed reporter in AAVS1 locus of HN10 hESCs line (HN10/BMI1) (Supplementary Fig. 2a−d). Consistently, HN10/BMI1 showed no significant upregulation of the known naïve pluripotency markers (Supplementary Fig. 2e), but significantly enhanced antiapoptosis and survival rate upon injection into mouse later morulas or early blastocysts (Supplementary Fig. 2f–g). Furthermore, 7.5% of recovered E10.5 mouse embryos injected with HN10/BMI1 were detected to have human cell chimeras, using 1/10^4^ as a threshold (Fig. 1j, Supplementary Fig. 2h). In total, human cells could be detected in 9.52% of the recovered mouse embryos injected with BMI1-expressed UH10 or HN10 hPSCs. Moreover, we found that BMI1 would be no longer required for chimera formation once hPSCs survived through the initial stage in the early embryos (Supplementary Fig. 3a–b).

**Fig. 2:**
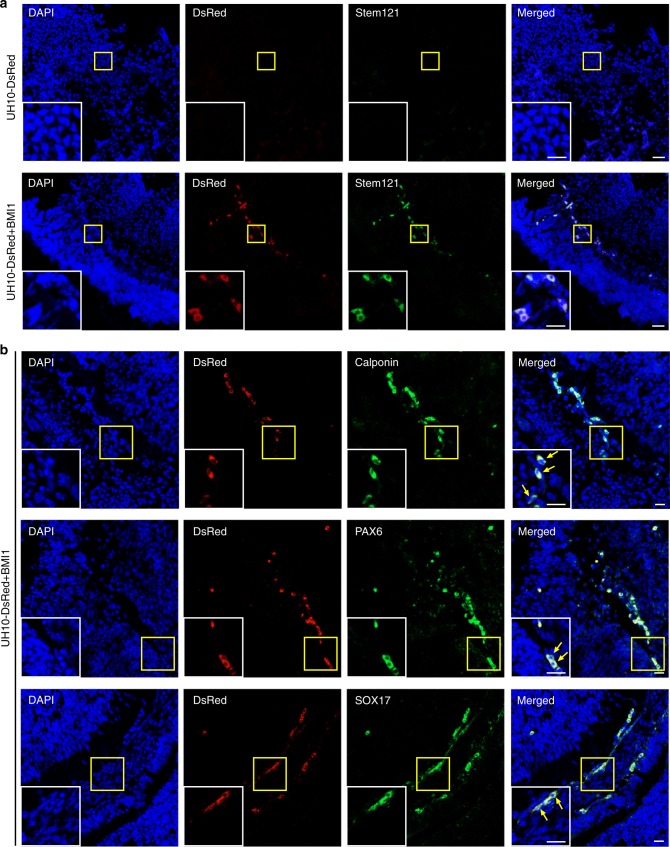
Contribution of different lineage cells by hPSCs + BMI1. Sections of E10.5 mouse chimeric embryos were analyzed by immunostaining on selected markers. **a** Representative images showing the integrated DsRed^+^ human cells coexpress human-specific marker Stem121 in chimeric embryos. Scale bars, 20 µm (left), 50 µm (right). **b** DsRed^+^ human cells in mouse chimeric embryo were costained with antibodies against Calponin, mesoderm marker, PAX6, ectoderm marker and SOX17, endoderm marker. The insets are the zoom-in pictures of the areas of yellow box. Scale bars, 20 µm (left), 50 µm (right)

To exclude the possibility that human DNA might be derived from lysed or dead cells^23^, we further performed immunostaining on sections of E10.5 mouse embryos injected with UH10-DsRed or UH10-DsRed + BMI1 cells. DsRed^+^ cells, costaining with Stem121, a human-specific antibody, could be clearly detected in E10.5 embryos injected with UH10-DsRed + BMI1 cells, while not UH10-DsRed cells (Fig. 2a). Furthermore, these DsRed^+^ human cells were also costained with antibodies against markers of different lineages, for example, PAX6, an ectoderm marker^28^, Calponin, a mesoderm marker^29^, and SOX17, an endoderm marker^30^ (Fig. 2b). These data indicate that the survived hiPSCs underwent differentiation into different lineages in mouse chimera. In sum, these data demonstrate that BMI1 enables the conventional hPSCs to survive and integrate into preimplantation mouse morulas or blastocysts and subsequently contribute to chimera in postimplantation embryos.

### BMI1-expressed hPSCs contribute to extra-embryonic tissues

**Fig. 3:**
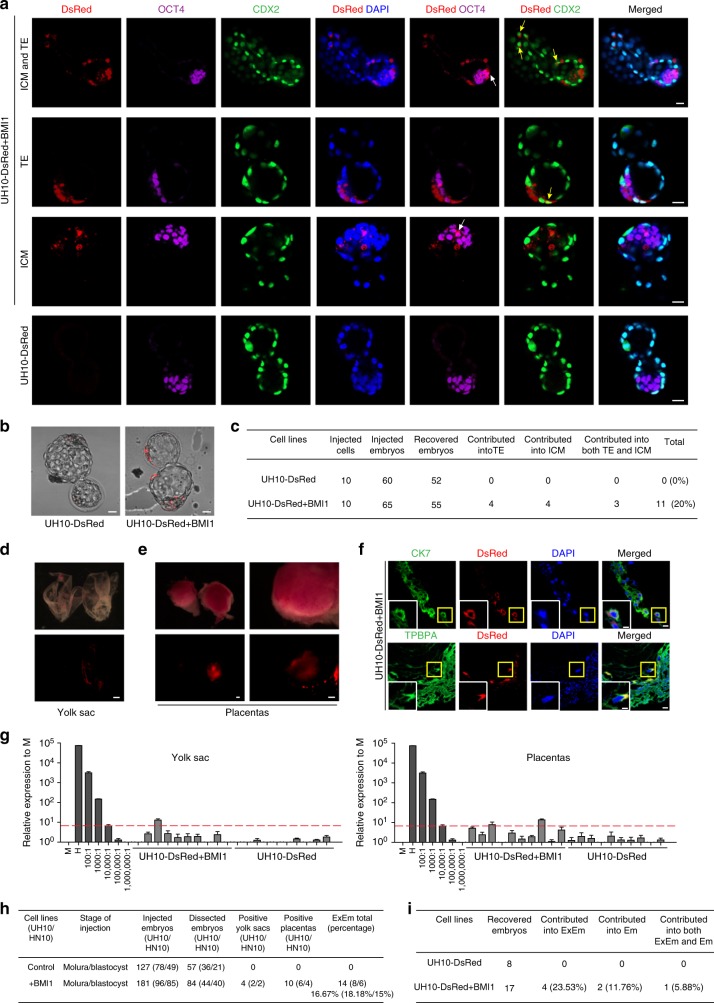
Contribution of extra-embryonic tissues by hPSCs + BMI1. **a**−**c** Ten indicated DsRed^+^ cells were microinjected into 8C stage mouse embryos and analyzed after 48–60 h culture in vitro. **a** Representative images showing the integrated UH10-DsRed^+^ with BMI1 expression coexpress OCT4, ICM marker or CDX2, early trophoblast marker in cultured chimeric embryos. White arrow, DsRed^+^/OCT4^+^ cells; yellow arrow, DsRed^+^/CDX2^+^ cells; scale bars, 20 µm. **b** Images of blastocysts injected with ten indicated cells after 48–60 h culture in vitro; scale bars, 20 µm. **c** Summary of chimera assays with injection of ten indicated DsRed^+^ cells at the 8C stage embryo, and followed 48–60 h in vitro development into blastocyst stage. **d**−**f** Contribution of extra-embryonic tissues by the injected hPSCs in E10.5 mouse embryos. **d** Representative images of the E10.5 chemaric yolk sac with injection of the UH10-DsRed + BMI1 cells in later morulas or early blastocysts; scale bars, 1 mm. **e** Images of the E10.5 chimeric placentas with injection of the UH10-DsRed + BMI1 cells in later morulas or early blastocysts; scale bars, 1 mm. **f** Representative placenta confocal images showing DsRed^+^ human cells can contribute to trophoblastic lineages in chimeric E10.5 placentas. The placentas were stained with anti-CK7 and TPBPA. The bottom images are the zoom-in pictures of the areas of yellow box; scale bars, 50 µm (up), 25 µm (down). **g** Representive quantitative genomic PCR analysis of the human mitochondria DNA in E10.5 mouse yolk sacs and placentas after injection of the indicated cells; **h** Summary of positive yolk sacs and placentas. **i** Summary of contribution of both ExEm and Em in in vivo chimera experiments. Embryos were recovered at E10.5 stage. ExEm extra-embryonic tissues, Em embryos

It has been well documented that most PSCs cultured in vitro, even the naïve-state mouse PSCs show limited contributions to extra-embryonic tissues (ExEm) in vivo^31^. Recent report showed that two new types of PSCs, derived with the aid of specific chemical cocktail retained the competency to form ExEm chimera^32,33^. However, whether the conventional hPSCs could contribute ExEm in vivo remains questionable, even though they were shown to differentiate into trophoblast cells in vitro^34,35^. To address this question, we firstly injected 10 UH10-DsRed and UH10-DsRed + BMI1 cells respectively into eight-cell (8C) stage mouse embryos and examined ExEm chimera after culture for 48–60 h in vitro. The ExEm and inner cell mass (ICM) chimera were examined by coimmunostaining on the trophoblast marker (CDX2) and ICM marker (OCT4)^33^ (Fig. 3a). As expected, we failed to detect efficient contribution of human cells in embryos injected with UH10-DsRed cells (Fig. 3a–c). In contrast, the DsRed^+^ cells costained with CDX2 or OCT4 could be detected in embryos injected with UH10-DsRed + BMI1 cells (Fig. 3a–c). Totally, we observed that 20% of the recovered embryos at blastocyst stage injected with UH10-DsRed + BMI1 cells showed chimeras with ICM, trophoblast or both (Fig. 3a–c).

We then examined the contribution of ExEm chimera with UH10-DsRed + BMI1 cells in vivo. Certain number of the E10.5 embryos injected with UH10-DsRed + BMI1 cells showed chimera with DsRed^+^ cells in ExEm tissue, such as placenta and yolk sac (Fig. 3d, e). Moreover, the DsRed^+^ cells in ExEm tissue could be costained with antibody against CK7 or TPBPA, two known trophoblast markers^33^, demonstrating the contribution of hPSCs into ExEm lineages (Fig. 3f). Based on human mitochondrial PCR assay, we further confirmed that the UH10-DsRed + BMI1 cells could contribute to yolk sac and placentas in vivo (Fig. 3g, h, Supplementary Fig. 4a−b), so as the HN10 + BMI1 cells (Fig. 3h, Supplementary Fig. 4c−d). In sum, human cells could be detected in 16.67% of the recovered mouse yolk sac/plancentas injected with BMI1-expressed UH10 (18.18%) or HN10 (15%) hPSCs. Again, BMI1 was no longer required for ExEm contribution once hPSCs survived through the initial stage in the embryos (Supplementary Fig. 3a−b). Interestingly, we note that the hPSC contribution to both Em and ExEm lineages is much lower than that to either Em or ExEm alone based on both the in vitro and in vivo data (Fig. 3c–i), indicating the embryonic contribution of hPSCs appears to be random.

### BMI1-expressed hPSCs integrate into rabbit and pig embryos

**Fig. 4:**
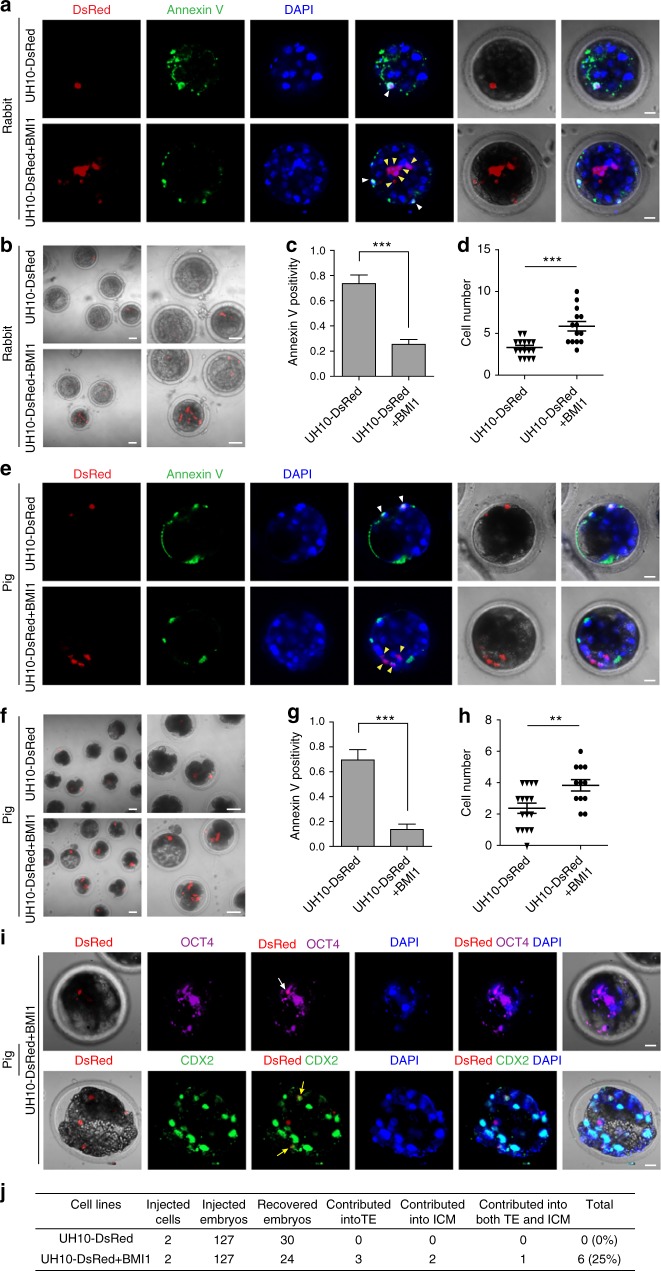
hiPSCs + BMI1 integrate into rabbit and pig preimplantation blastocyst. **a**−**d** Integration of hiPSCs + BMI1 in rabbit blastocysts. **a** Representative images of rabbit embryos stained with Annexin V after injection of seven indicated DsRed^+^ cells in blastocysts and 1-day culture in vitro. White arrow, DsRed^+^/Annexin V^+^ cells; Yellow arrow, DsRed^+^/Annexin V^−^ cells. Scale bars, 20 µm. **b** Images of rabbit blastocysts injected with seven indicated cells after 1-day culture in vitro, scale bars, 50 µm (left), 20 µm (right). **c** Statistics of the percentage of engrafted DsRed^+^ cells that were Annexin V^+^ in the injected rabbit blastocysts; mean + SEM of 16 (UH10-DsRed) or 14 (UH10-DsRed + BMI1) samples, ***p < 0.001. **d** Statistics of the DsRed^+^ cells in the injected rabbit blastocysts; mean ± SEM of 16 (UH10-DsRed) or 14 (UH10-DsRed + BMI1) samples, ***p < 0.001. **e**−**h** Integration of hiPSCs + BMI1 in pig blastocysts. **e** Representative images of pig embryos stained with Annexin V after injection of 3–5 indicated DsRed^+^ cells in later morulas or early blastocysts for 1-day culture in vitro. White arrow, DsRed^+^/Annexin V^+^ cells; Yellow arrow, DsRed^+^/Annexin V^−^ cells. Scale bars, 20 µm. **f** Images of pig blastocysts injected with the indicated cells after in vitro culture. Scale bars, 50 µm (left), 20 µm (right). **g** Statistics of the percentage of engrafted DsRed^+^ cells with Annexin V^+^ in injected pig blastocysts; mean + SEM of 16 (UH10-DsRed) or 12 (UH10-DsRed + BMI1) samples, ***p < 0.001. **h** Statistics of the number of DsRed^+^ cells injected in pig blastocysts; mean ± SEM of 16 (UH10-DsRed) or 12 (UH10-DsRed + BMI1) samples, **p < 0.01. **i** Representative images of pig blastocyst injected with UH10-DsRed + BMI1 stained with antibodies against the indicated markers. Two indicated DsRed^+^ cells were injected to 8C stage pig embryos. The integrated UH10-DsRed^+^ with BMI1 expression coexpress OCT4 or CDX2 in cultured chimeric embryos after additional 4 days’ culture in vitro. White arrow, DsRed^+^/OCT4^+^ cells; yellow arrow, DsRed^+^/CDX2^+^ cells. Scale bars, 20 µm. **j** Summary of chimera assays injected with the indicated DsRed^+^ cells at the 8C stage pig embryo, and the injected embryos were cultured for additional 4 days to blastocysts

We then examined whether BMI1 could enable hPSCs to integrate and form chimera in preimplantation blastocyst of alternative species. Firstly, we injected seven UH10-DsRed and UH10-DsRed + BMI1 cells respectively into the rabbit blastocysts. Again, we found that only UH10-DsRed + BMI1 cells showed significant resistance to apoptosis in rabbit embryos, and consistently, the number of survived UH10-DsRed + BMI1 cells were much higher compared with UH10-DsRed cells (Fig. 4a–d). Similarly, UH10-DsRed + BMI1 cells also showed much more antiapoptosis in pig later morulas or early blastocysts compared with UH10-DsRed cells (Fig. 4e–h). Furthermore, we found that the integrated DsRed^+^ human cells in pig embryos could be costained with either OCT4 or CDX2 after in vitro culture, indicating the contribution to both ICM and ExEm in cultured pig embryos (Fig. 4i, j). Together, these data demonstrate that overcoming apoptosis by BMI1 allows the conventional hPSCs to integrate into preimplantation morulas or blastocysts of multiple species.

### Antiapoptosis indicates interspecies chimera in naive hPSCs

**Fig. 5:**
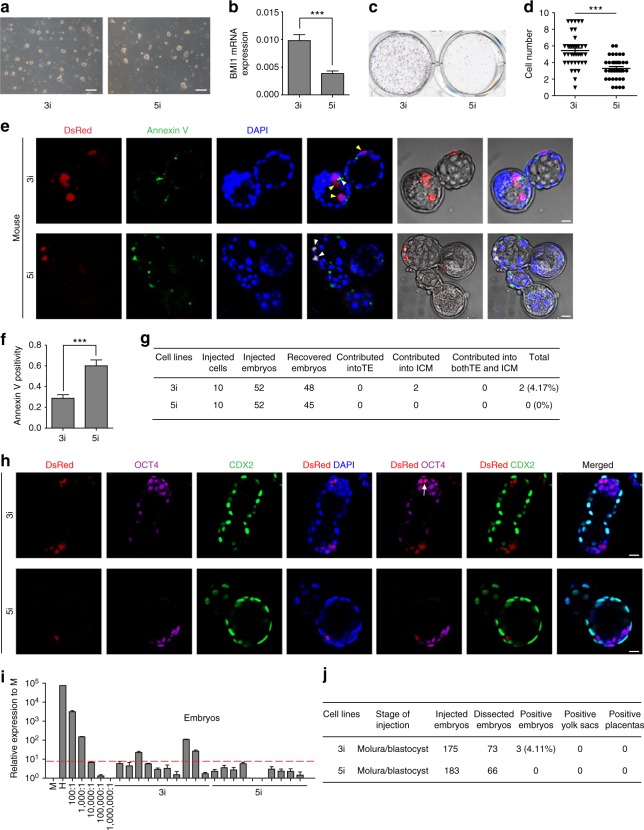
Antiapoptosis enables interspecies chimera with naive hPSCs. **a** Phase contrast of 3i and 5i naïve cells converted from primed UH10-DsRed hiPSCs; scale bars, 100 µm. **b** RT-qPCR analysis of BMI1 expression in the indicated cells; mean + SEM of three independent samples, ***p < 0.001. **c** Alkaline phosphatase staining on colonies formed by the indicated cells after plating of 50,000 cells on feeder for 7 days. **d** Statistics of the numbers of the indicated DsRed + cells in injected mouse blastocysts after injection of the ten indicated DsRed + cells in later morulas or early blastocysts and 1-day culture in vitro; scale bars, 50 µm; mean ± SEM of 40 (3i) or 37 (5i) samples, ***p < 0.001. **e** Representative fluorescence images of mouse embryos stained with Annexin V after injection of ten indicated DsRed^+^ cells in later morulas or early blastocysts and 1-day culture in vitro. White arrow, DsRed^+^/Annexin V^+^ cells; Yellow arrow, DsRed^+^/Annexin V^−^ cells. Scale bars, 20 µm. **f** Statistics of the percentage of the engrafted DsRed+ cells with Annexin V+ in the mouse blastocysts; mean + SEM of 40 (3i) or 37 (5i) samples, ***p < 0.001. **g** Summary of chimera assays with injection of ten indicated DsRed^+^ cells at the 8C stage embryo, and followed 48–60 h in vitro development into blastocyst stage. **h** Representative images showing the integration of indicated cells coexpressed OCT4, ICM marker or CDX2, early trophoblast marker in cultured chimeric embryos. White arrow, DsRed^+^/OCT4^+^ cells; scale bars, 20 µm. **i** Representative quantitative genomic PCR analysis of the human mitochondria DNA in E10.5 mouse embryos after injection of the indicated cells. A human DNA control (H), a mouse DNA control (M) and a series of human−mouse cell dilutions (1/100 to 1/1000,000) were run in parallel to estimate the degree of human cell integration. The dashed line indicates the detection level of one human cell in 10,000 mouse cells. **j** Summary of chimera assays of the indicated cells injectied at the later morula or early blastocyst stage

It is shown that the naïve-like hPSCs also showed limited interspecies chimerism, even though they are supposed to match the developmental stage^23^. We then examined whether the initial apoptosis also happens in naïve hPSCs injected into mouse embryos. Firstly, we generated naïve-like hPSCs from UH10 hPSCs based on two published protocols, the 3i protocol^20^ and 5i protocol^22^. Both the 3ihPSCs and 5ihPSCs shared typical naïve-like characteristics (Fig. 5a and Supplementary Fig. 5a). However, 3i hPSCs showed much higher BMI1 expression compared with 5i hPSCs (Fig. 5b). Furthermore, when plated at single-cell density, 3i hPSCs showed much better cloning efficiency and less apoptosis compared with 5i hPSCs (Fig. 5c−f). 3i hPSCs also showed much better antiapoptosis and survival rate than 5i hPSCs when injected into the mouse embryos (Fig. 5e, f). Consistently, we could detect certain number of cultured embryos had ICM contribution by 3i hPSCs, while not 5i hPSCs (Fig. 5h−g). Moreover, 3i hPSC-derived chimera could be detected in lower number of mouse postimplantation conceptuses (Fig. 5i, j). In contrast, no contribution of 5i hPSCs could be detected in the similar number of analyzed mouse embryos (Fig. 5i, j). Together, these data demonstrate that the antiapoptosis is also an essential factor to ensure interspecies chimerism with human naïve PSCs.

## Discussion

It has been well documented that hPSCs exhibit very limited efficiency in forming preimplantation interspecies chimeras^9,12^. Uncovering the barriers and mechanisms underlying interspecies chimerism using hPSCs is of both conceptual and practical importance. One explanation is that the conventional hPSCs represent the primed state, rather the naïve one, and thus are not able to form preimplantation chimeras^14^. However, it is still hard to obtain significantly increased efficiency in interspecies chimera using the some types of the converted naïve hPSCs^9,23^, indicating that other barriers remain to be uncovered. Recently, a new type of hPSCs, termed extended pluripotent stem cells (EPS), deriving with the aid of a chemical cocktail, were reported to show interspecies chimerism in mouse preimplantation blastocyst^33^. Interestingly, these mouse or human EPS cells exhibit distinct expression profiles compared with the known naïve-state PSCs^33^. The precise mechanisms that confer these human EPS cells with the competency to form chimeras remain unclear. To obtain a successful interspecies chimera, both the hospitality of the host embryo and competency of the introduced PSCs are essential issues to be considered. The conventional hPSCs, despite their distinct characteristics to naïve mESCs, are theoretically pluripotent as they could form teratomas consisting of three typical embryonic germ layers^11^ as well as differentiate into trophoblast in vitro^34,35^. Therefore, the conventional hPSCs might in theory hold the potential to differentiate in host embryos if they could integrate and survive well in preimplantation morulas or blastocysts.

Here, we show that hPSCs underwent apoptosis and disappeared rapidly when injected into preimplantation embryos of multiple species including mouse, rabbit, and pig. Our findings suggest that cell apoptosis is the first and general barrier in interspecies chimerism using hPSCs, which is consistent to previous findings in mouse EpiSCs^10^. We have previously shown that BMI1, a polycomb factor, could overcome the individualization-induced apoptosis in hPSCs through suppressing P16^Ink4a^ and P14^ARF^ pathways and allow hPSCs to proliferate on gelatin, a nonbioactive and inhospitable matrix^26^. However, known naïve markers and other phenotypes were not significantly changed in hPSCs with BMI1 expression^26^. Here in this study, BMI1 also overcomes cell apoptosis in hPSCs when introduced into another inhospitable environment, the morulas or blastocysts of different species. Interestingly, these survived hPSCs in preimplantation embryos could contribute various lineages in subsequent embryonic development in mouse embryos. Strikingly, contributions to the extra-embryonic lineages like, placenta, yolk sac by hPSCs could be clearly observed. Interestingly, hPSCs cultured under EPS conditions also displayed elevated single-cell survival rate in vitro^33^, indicating the antiapoptosis is a general property for cells that could form interspecies chimera. Indeed, we observed that the naïve hPSCs with higher level of BMI1 expression and better antiapoptosis, otherwise did not, showed higher efficiency of interspecies chimera formation. Notably, while this manuscript was under review, another report published that hESCs could contribute to chimeras in mouse embryos with the aid of BCL2-mediated antiapoptosis^36^. These reports and together with ours here suggest that the initial apoptosis is a major barrier in interspecies chimerism using hPSCs. Further studies need to uncover the blocks after implantation to further enhance the efficiency of interspecies chimerism using hPSCs.

## Methods

### Culture and maintenance of hiPSCs and hESCs

Human PSC line UH10/HN10-DsRed and UH10/HN10-DsRed + BMI were maintained on Matrigel-coated plates with hPSCs medium mTeSR1 (STEM CELL) and passaged every 3 days using 0.5 mM ethylenediaminetetraacetic acid disodium salt (EDTA-2Na). Cells were cultured under 20% O_2_ and 5% CO_2_ at 37 °C condition. All of the cell lines indicated above have been tested to be free of mycoplasma contamination. The hESC line HN10 was a gift from our collaborative colleagues in Hainan Medical University, generated by a whole-mechanical method^27^. In particular, the generation of the UH10 hiPSCs and HN10 hESCs was approved by the Institutional Review Board at Guangzhou Institutes of Biomedicine and Health, and Hainan Medical University, respectively. Also, we have complied with all relevant ethical regulations and obtained consents from all human participants.

### Generation of DsRed-labeled hPSCs

To construct UH10 hiPSCs and HN10 hESCs with constitutive expression of DsRed in AAVS1 habor locus, guide RNA (gRNA) for AAVS1 safe harbor locus was designed on the website (crispr.mit.edu) and cloned into pX330 (a vector that can express Cas9 protein and guide RNA). Donor plasmid (pUC57-Neomycin-AAVS1-CAG-DsRed) contained left and right homology arms of AAVS1 safe harbor locus. 1 × 10^6^ hPSCs were electroporated with 4 μg of donor plasmid and 2 μg of pX330 plasmid and DsRed^+^ cells were primarily selected by G418 (100 μg per mL) and then sorted by FACS (fluorescence-activated cell sorting).

### Generation of BMI1 forced-expression hPSCs

The human BMI1 gene was cloned into a lentiviral vector tetO-FUW for tet-inducible expression of BMI1. Lentivirus was produced in 293T cells by cotransfecting the tetO-FUW-BMI1 with three helper plasmids (pRSV-REV, pMDLg/pRRE, and vesicular stomatitis virus G protein expression vector), which provide the essential elements to package lentivirus^37^. Viral supernatants were collected at 48 h after transfection and passed through a 0.45 µm filter to remove cell debris, then subjected to ultracentrifugation (20,000 × *g* for 3 h at 4 °C). DsRed^+^ hPSCs were transduced with tetO-FUW-BMI1 lentivirus. The expression of BMI1 is induced by exogenous addition of doxycycline (DOX) (2 μg per mL) and the positive cells were selected by puromycin (1 μg per mL).

### Generation of naïve hPSCs

For 3i naïve hPSCs, we used the methods reported by Austin Smith’s lab to induce primed human PSCs into naïve human PSCs^20^. KLF2 and NANOG were dox inducible overexpressed in hPSCs by tetO-FUW lentivirus as we described above. hPSCs with KLF2/NANOG expression were further transferred into switched medium (N2B27 + 2iL medium: 50% DMEM/F12 (Hyclone), N2 (Gibco, 200×), B27 (Gibco, 100×), Glutamax (Gibco, 200×), 50% Neurobasal (Gibco), Sodium Pyruvate (Gibco, 100×), NEAA (Gibco, 200×), 3 μM CHIR99021 (Selleck), 1 μM PD0325901 (Selleck), 100 μM β-mercaptoethanol (gibco), 10 ng per mL human LIF) in the presence of DOX to induce transgene expression. These cells were cultured on mitomycin C-inactivated MEF (ICR mouse embryonic fibroblasts) feeder in N2B27 + 2iL  +  Dox medium and passaged every week. After 3–4 passages, these naïve cells were cultured on feeder in N2B27 + 2iL + Gö6983 (2 μM) medium without Dox-inducible transgene expression.

For 5i naïve hPSCs, we used the methods reported by Rudolf Jaenisch’s lab to convert primed state hPSCs into naïve state^22^. Briefly, 2×10^5^ hPSCs were seeded on mitomycin C-inactivated MEF feeder and cultured in KSR medium (85% DMEM/F12 (Hyclone), 15% KSR (Gibco), Glutamax (Gibco, 100×), NEAA (Gibco, 100×), β-mercaptoethanol (gibco, 1000×), and 8 ng per mL bFGF (R&D Systems), 10 μM Y-27632 (Sigma)) for 4 days. Then these cells were passaged and cultured on mitomycin C-inactivated feeder in 5i/L medium (50% DMEM/F12 (Hyclone), 50% Neurobasal (Gibco), N2 (Gibco, 200×), B27 (Gibco, 100×), Glutamax (Gibco, 100×), NEAA (Gibco, 100×), 50 μg per mL BSA (Sigma), β-mercaptoethanol (gibco, 1000×), 1 μM PD0325901 (Selleck), 1 μM IM-12 (Enzo), 0.5 μM SB590885 (R&D Systems), 1 μM WH-4-023 (A Chemtek), 10 μM Y-27632 (Sigma), 20 ng per mL human LIF (Pepretech)) for 10 days. The 5i cells were passaged every 5 days.

### Animal experiments

The interspecies chimerism experiments using hPSCs have been approved by the Ethnical Committee on Animal Experiments at Guangzhou Institutes of Biomedicine and Health, Chinese Academy of Sciences. Also, all the experiments were performed according to the ISSCR guidelines.

ICR mice were purchased from Beijing Vital River Laboratory Animal Technology Co., Ltd. Female rabbits at 4–6 weeks of age were selected as donors or surrogate mother. Two days before mating the donors were injected with 7.5 U pregnant mare’s serum gonadotropin (PMSG), and 48 h later the donors were injected with 7.5 U hCG and mated with the male mouse. The eight-cell stage embyos and late morulas and early blastocysts were obtained at E1.5 and E3.5, respectively. Ten cells were injected into the embryos for following experiments. For in vitro apoptosis and chimerism assay, the embryo culture medium—KSOM (Millipore) was added with dox (2 μg per mL) for inducible BMI1 expression. For in vivo chimera assay, the 10–20 embryos were transplanted into the uterus of per pseudopregnant mouse and the surrogate mice with embryos injected with BMI1 force-expressed hPSCs were fed with the dox-containing water (2 mg per mL) until E10.5, otherwise indicated.

New Zealand rabbits were obtained from Huadong Xinhua experimental animal farms, District of Guangzhou. Female rabbits at 4–6 weeks of age were used as donors and injected with 100 IU PMSG, and 72–120 h later further injected with 100 IU hCG and mated with the male rabbits. Embryos at late morula and early blastocyst were harvested 4–5 days after hCG injection, injected with seven indicated cells for in vitro assay, and cultured in Earle's balanced salt solution (EBSS, Thermo) Medium.

Pigs were purchased from a local slaughterhouse and cumulus oocyte complexes were aspirated from antral follicles. After 44 h in vitro maturation, the oocytes were activated with two successive DC pulses of 120 V per mm for 30 μs using an electrofusion instrument (CF-150B, BLS, Hungary). The activated oocytes were cultured in PZM-3 medium for partheno-development. After 48 h or 4 days of culture, embryos at eight-cell stage or later morula to early blastocyst stage were selected respectively for microinjection. Two indicated cells were injected to eight-cell stage embryos and 3–5 cells were injected to later morulas or early blastocysts embryos. The injected embryos were cultured in PZM3 medium (ENZO) for further analysis.

### Apoptosis assays

hPSCs were completely dissociated using Accutase and centrifuged at 300 × *g* at room temperature for 3 min. After removing the supernatant, cells were resuspended in the culture medium at a proper density (2–6 × 10^5^ cells per mL) and placed on ice for 20–30 min before injection. Cells were injected into mouse, rabbit, and pig later morulas or early blastocysts cultured in vitro for 24 h. Then, embryos were fixed with 4% paraformaldehyde (PFA) for 30 min and incubated with Annexin V for overnight at 4 °C. After washing with phosphate buffered saline (PBS) for three times, embryos were stained with 4',6-diamidino-2-phenylindole (DAPI). Stained embryos were observed using a LSM800 confocal microscope (Carl Zeiss).

### Chimerism analysis

For chimerism analysis, cells were dissociated into single cells using Accutase and centrifuged at 300 × *g* at room temperature for 3 min. After removal of the supernatant, cells were resuspended in the culture medium at a proper density (2–6 × 10^5^ cells per mL) and placed on ice for 20–30 min before injection. For in vitro extra-embryonic chimerism analysis, cells were microinjected into the eight-cell stage embryos, while for in vivo chimerism analysis, cells were microinjected into the later morulas or early blastocysts. For in vitro chimerism assay, the injected embryos at blastocyst stage were fixed by 4% PFA for 30 min and immunostained (CDX2, OCT4, and DAPI). For chimerism analysis in vivo, embryos were harvested until E10.5 for human mitochondria DNA assay or immunostaining.

### Single-cell cloning efficiency

For single-cell cloning assay of primed hPSCs, cells were plated onto six-well plate at a density of 2000 cells per well, after dissociating into single cells with Accutase and centrifuged at 300 × *g* at room temperature for 3 min. Seven days later, cells were fixed in 4% PFA for 2 min. After washing with PBS, cells were stained with alkaline phosphatase staining solution (Beyotime) for 10−15 min.

For single-cell cloning assay of naïve hPSCs, cells were plated on mitomycin C-inactivated MEF (ICR mouse embryonic fibroblasts) feeder at a density of 50,000 cells per well of 6-well-plate, after dissociating into single cells as described above; also the colonies were stained accordingly.

### Quantitative genomic PCR assay

A highly sensitive mitochondrial PCR assay was used to quantitatively analyze the degree of integration of human cells in mouse conceptuses according to a previous publication^23^. Genomic DNA was extracted from E10.5-injected mouse embryos, yolk sacs and placentas. Twenty-five nanograms of genomic DNA per sample was used for qPCR analysis to measure the presence of human DNA. Primer sequences were listed in Supplementary Table 1.

### Immunofluorescence

The injected blastocyst embryos and frozen sections of E10.5 mouse chimeric embryos and placenta were incubated with antibodies (Stem121, Calponin, PAX6, OCT4, CK7, CDX2, and TPBPA) overnight at 4 °C, washed with PBS and incubated with specific secondary antibody. Nuclei were stained with DAPI. Stained embryos and sections were observed using a LSM800 confocal microscope (Carl Zeiss). Antibodies were listed in Supplementary Table 2.

### Western blot assays

Cells were lysed on ice with 200 µL of RIPA buffer (Beyotime) for 15 min and separated by 12% sodium dodecyl sulfate–polyacrylamide gel electrophoresis (SDS-PAGE) before being transferred onto polyvinylidene difluoride (PVDF) membranes (Millipore). The membranes were blocked in 5% nonfat milk for 1 h and incubated overnight at 4 ℃ with the appropriate diluted primary antibodies or anti-flag/GAPDH antibody. Subsequently, the membranes were incubated with HRP-conjugated secondary antibody for 2 h at room temperature and HRP was detected by ECL (Advanste) and visualized by SmatChemi Image Analysis System (SAGECREATION). Antibodies were listed in Supplementary Table 2.

### Quantitative real-time PCR

Total RNA was extracted with the RaPure Total RNA Micro Kit (Magen). Two-microgram RNA was reverse transcribed into cDNA and amplified with SYBR Green PCR Master Mix (Bio-Rad). Primer sequences were listed in Supplementary Table 1.

### Teratoma formation

The teratoma formation experiments were approved by the Ethical Committee on Animal Experiments at Guangzhou Institutes of Biomedicine and Health, Chinese Academy of Sciences. Cells were digested by Accuatse (Sigma) for 5 min at 37 °C and resuspended in 30% matrigel (Corning) in DMEM/F12 (Hyclone), and then injected subcutaneously into NOD/SCID immuno-deficient mice, obtained from Beijing Vital River Laboratory Animal Technology Co., Ltd. Teratomas were detected after 8 weeks and fixed in 4% PFA. After paraffin embedding and sectioning, sections were stained with hematoxylin/eosin.

### Statistics

In general, data were presented as mean + SEM, and statistics were determined by unpaired two-tailed Student’s test (*t* test). *p* value < 0.05 was considered statistically significant. *p < 0.05; **p < 0.01; ***p < 0.001. No statistical method was used to predetermine the sample size. No samples were excluded for any analysis. No randomization was used for allocating animal group. No blinding done in animal experiments.

## Electronic supplementary material

Supplementary Information

## Data Availability

The datasets generated and/or analyzed during the current study are available from the corresponding author on reasonable request.

## References

[1] Evans MJ, Kaufman MH (1981). Establishment in culture of pluripotential cells from mouse embryos. Nature.

[2] Takahashi K, Yamanaka S (2006). Induction of pluripotent stem cells from mouse embryonic and adult fibroblast cultures by defined factors. Cell.

[3] Tam PPL (2003). Mouse embryonic chimeras: tools for studying mammalian development. Development.

[4] Mascetti VL, Pedersen RA (2016). Contributions of mammalian chimeras to pluripotent stem cell research. Cell Stem Cell.

[5] Wu J (2017). Interspecies chimerism with mammalian pluripotent stem cells. Cell.

[6] Brons IG (2007). Derivation of pluripotent epiblast stem cells from mammalian embryos. Nature.

[7] Huang Y, Osorno R, Tsakiridis A, Wilson V (2012). In Vivo differentiation potential of epiblast stem cells revealed by chimeric embryo formation. Cell Rep..

[8] Kojima Y (2014). The transcriptional and functional properties of mouse epiblast stem cells resemble the anterior primitive streak. Cell Stem Cell.

[9] Masaki H (2015). Interspecific in vitro assay for the chimera-forming ability of human pluripotent stem cells. Development.

[10] Masaki H (2016). Inhibition of apoptosis overcomes stage-related compatibility barriers to chimera formation in mouse embryos. Cell Stem Cell.

[11] Thomson JA (1998). Embryonic stem cell lines derived from human blastocysts. Science.

[12] James D, Noggle SA, Swigut T, Brivanlou AH (2006). Contribution of human embryonic stem cells to mouse blastocysts. Dev. Biol..

[13] Tesar PJ (2007). New cell lines from mouse epiblast share defining features with human embryonic stem cells. Nature.

[14] Mascetti VL, Pedersen RA (2016). Human-mouse chimerism validates human stem cell pluripotency. Cell Stem Cell.

[15] Gafni O (2013). Derivation of novel human ground state naive pluripotent stem cells. Nature.

[16] Buecker C (2010). A murine ESC-like state facilitates transgenesis and homologous recombination in human pluripotent stem cells. Cell Stem Cell.

[17] Chan YS (2013). Induction of a human pluripotent state with distinct regulatory circuitry that resembles preimplantation epiblast. Cell Stem Cell.

[18] Guo G (2016). Naive pluripotent stem cells derived directly from isolated cells of the human inner cell mass. Stem Cell Rep..

[19] Hanna J (2010). Human embryonic stem cells with biological and epigenetic characteristics similar to those of mouse ESCs. Proc. Natl. Acad. Sci. USA.

[20] Takashima Y (2014). Resetting transcription factor control circuitry toward ground-state pluripotency in human. Cell.

[21] Ware CB (2014). Derivation of naive human embryonic stem cells. Proc. Natl. Acad. Sci. USA.

[22] Theunissen TW (2014). Systematic identification of culture conditions for induction and maintenance of naive human pluripotency. Cell Stem Cell.

[23] Theunissen TW (2016). Molecular criteria for defining the naive human pluripotent state. Cell Stem Cell.

[24] Xue Y (2013). Generating a non-integrating human induced pluripotent stem cell bank from urine-derived cells. PLoS ONE.

[25] Li D (2016). Optimized approaches for generation of integration-free iPSCs from human urine-derived cells with small molecules and autologous feeder. Stem Cell Rep..

[26] Wang WQ (2017). Suppressing P16(Ink4a) and P14(ARF) pathways overcomes apoptosis in individualized human embryonic stem cells. FASEB J..

[27] Li B (2010). A whole-mechanical method to establish human embryonic stem cell line HN4 from discarded embryos. Cytotechnology.

[28] Zhang XQ (2010). Pax6 is a human neuroectoderm cell fate determinant. Cell Stem Cell.

[29] Vodyanik MA (2010). A mesoderm-derived precursor for mesenchymal stem and endothelial cells. Cell Stem Cell.

[30] Li QH (2017). A sequential EMT-MET mechanism drives the differentiation of human embryonic stem cells towards hepatocytes. Nat. Commun..

[31] De Los Angeles A (2015). Hallmarks of pluripotency. Nature.

[32] Yang J (2017). Establishment of mouse expanded potential stem cells. Nature.

[33] Yang Y (2017). Derivation of pluripotent stem cells with in vivo embryonic and extraembryonic potency. Cell.

[34] Horii M (2016). Human pluripotent stem cells as a model of trophoblast differentiation in both normal development and disease. Proc. Natl. Acad. Sci. USA.

[35] Xu RH (2003). BMP4 initiates human embryonic stem cell differentiation to trophoblast. Nat. Biotechnol..

[36] Wang X (2018). Human embryonic stem cells contribute to embryonic and extraembryonic lineages in mouse embryos upon inhibition of apoptosis. Cell Res..

[37] Zhang Y (2013). Rapid single-step induction of functional neurons from human pluripotent stem cells. Neuron.

