# Supplementary material for: BMI1 enables interspecies chimerism with human pluripotent stem cells
Source: Nat Commun. 2018 Nov 7;9:4649. doi: 10.1038/s41467-018-07098-w (PMC6220315; doi:10.1038/s41467-018-07098-w)
Supplement: Supplementary file 1 — Supplementary Information [file 41467_2018_7098_MOESM1_ESM.pdf]

Supplementary Figure 1

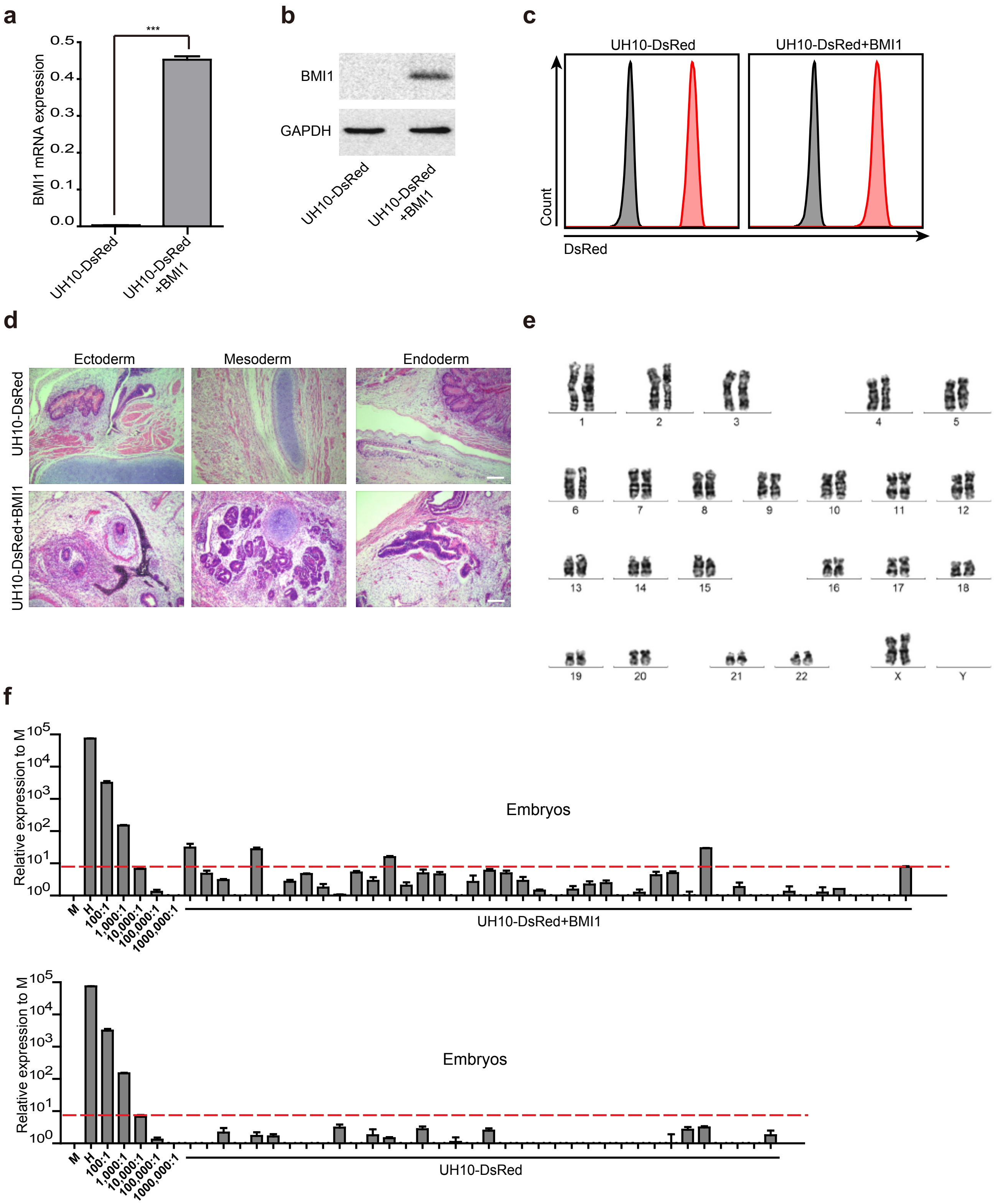

Supplementary Figure 1 Identification of BMI1 expression and pluripotency of UH10-DsRed+BMI1. (a-b) Quantitative PCR and western blot analysis of BMI1 expression of the indicated cells. Error bars represent mean + SEM of three independent replicates; ***p < 0.001. **(c)** FACS analysis of the DsRed fluorescence of the indicated cells. **(d)** Hematoxylin and eosin (HE) staining of the teratoma formed by UH10-DsRed and UH10-DsRed+BMI1+DOX (+DOX means mice were fed with Dox (2mg/ml) containing water). Scale bars, 100 µm. **(e)** Karyotype analysis of the UH10-DsRed+BMI1. **(f)** Quantitative genomic PCR analysis of the human mitochondria DNA in all recovered E10.5 mouse embryos after injection of the indicated cells.

Supplementary Figure 2

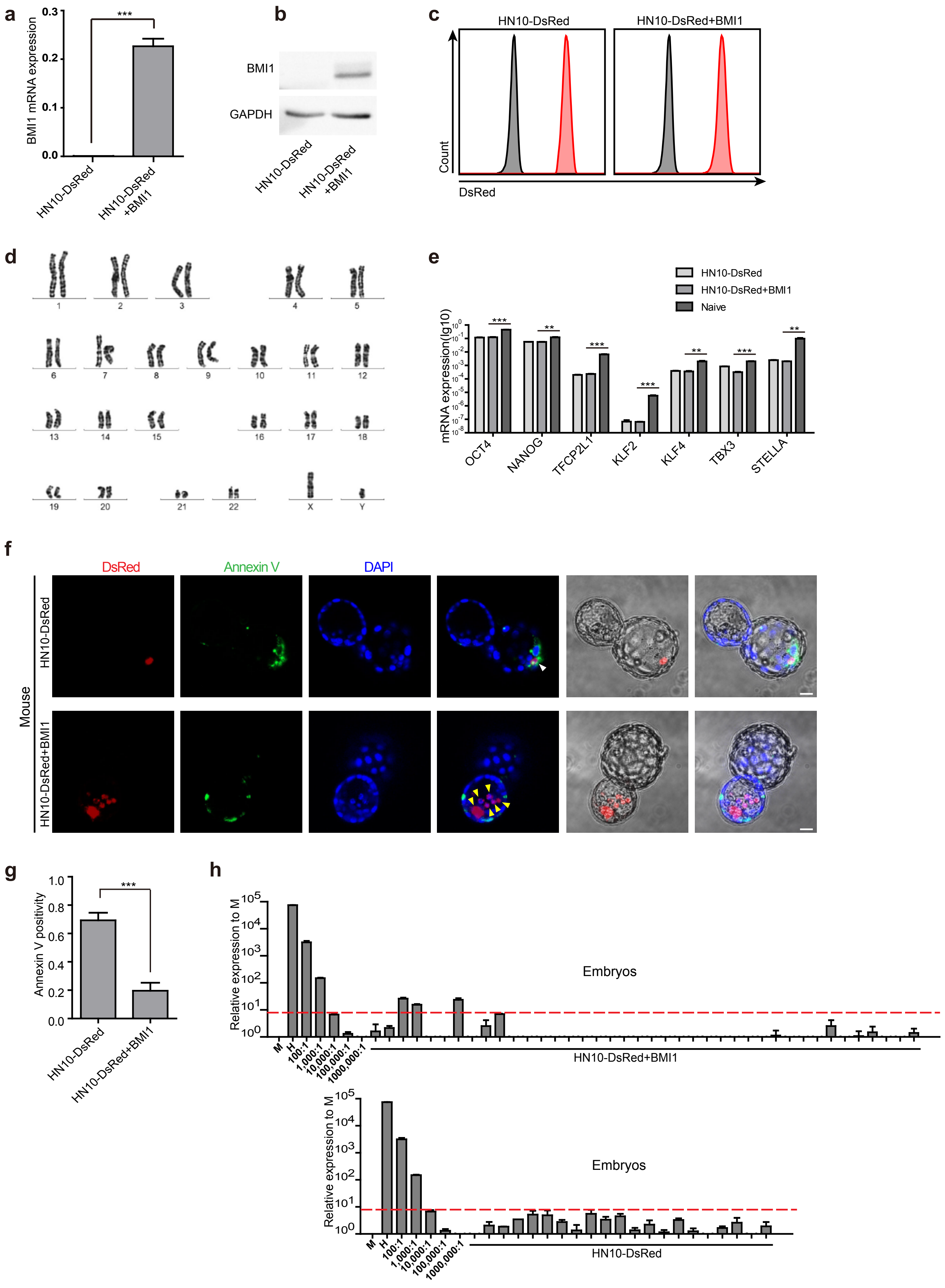

Supplementary Figure 2 BMI1 enables chimera formation with the HN10 hESCs. (a-b) Quantitative PCR and western blot analysis of BMI1 expression of HN10-DsRed and HN10-DsRed+BMI1. Error bars represent mean + SEM of three independent replicates; ***$p < 0.001$. **(c)** FACS analysis of the DsRed fluorescence of the indicated cells. **(d)** Karyotype analysis of HN10-DsRed+BMI1. **(e)** RT-qPCR analysis of the naïve markers expression of the indicated cells. Error bars represent mean + SEM of three independent replicates; **$p < 0.01$, ***$p < 0.001$. **(f)** Representative fluorescence images of mouse embryos stained with Annexin V after injection of 10 indicated $DsRed^+$ cells in later morulas or early blastocysts and 1-day culture *in vitro*. White arrow, $DsRed^+$/Annexin V^+ cells; Yellow arrow, $DsRed^+$/Annexin V^- cells. Scale bars, 20 µm. **(g)** Statistics of the percentage of engrafted $DsRed^+$ cells with Annexin V^+ in the mouse blastocysts; mean + SEM of 21 (HN10-DsRed) or 14 (NH10-DsRed+BMI1) samples, ***$p < 0.001$. **(h)** Quantitative genomic PCR analysis of the human mitochondria DNA in all recovered E10.5 mouse embryos after injection of the indicated cells.

Supplementary Figure 3

a

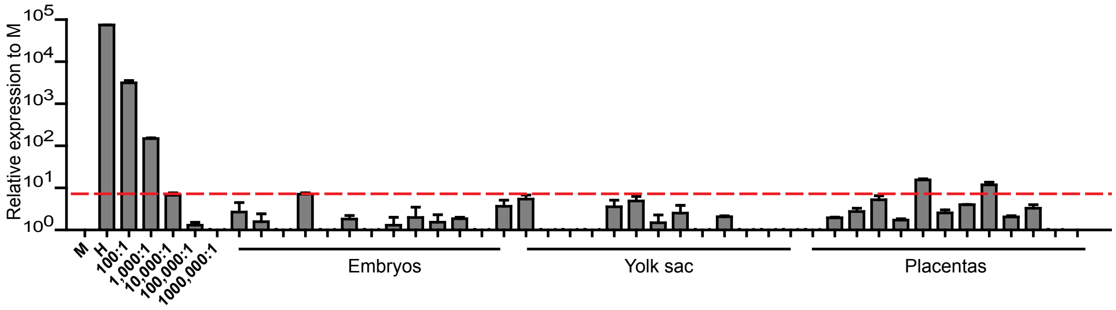

b

Cell lines	Stage of injection	Injected embryos	Dissected embryos	Positive embryos	Positive yolk sacs	Positive placentas	ExEm total	ExEm and Em
UH10-DsRed +BMI1-DOX	Morula/Blastocyst	34	13	1 (7.69%)	0 (0%)	2 (15.38%)	2 (15.38%)	0

Supplementary Figure 3 Temporal requirement for BMI1 in promoting chimera formation. (a) Quantitative genomic PCR analysis and summary of the human mitochondria DNA in E10.5 mouse embryos, yolk sacs and placentas after injection of the indicated cells (UH10-DsRed+BMI1-DOX). **(b)** Summary of chimera assays of the indicated DsRed^{+} cells injected at the later morula or early blastocyst stage. Addition of doxycycline in the water to the mouse surrogate mothers during day 0 to day 3 (at which point the transferred embryos developed to E6.5) after transfer of the indicated cells.

Supplementary Figure 4

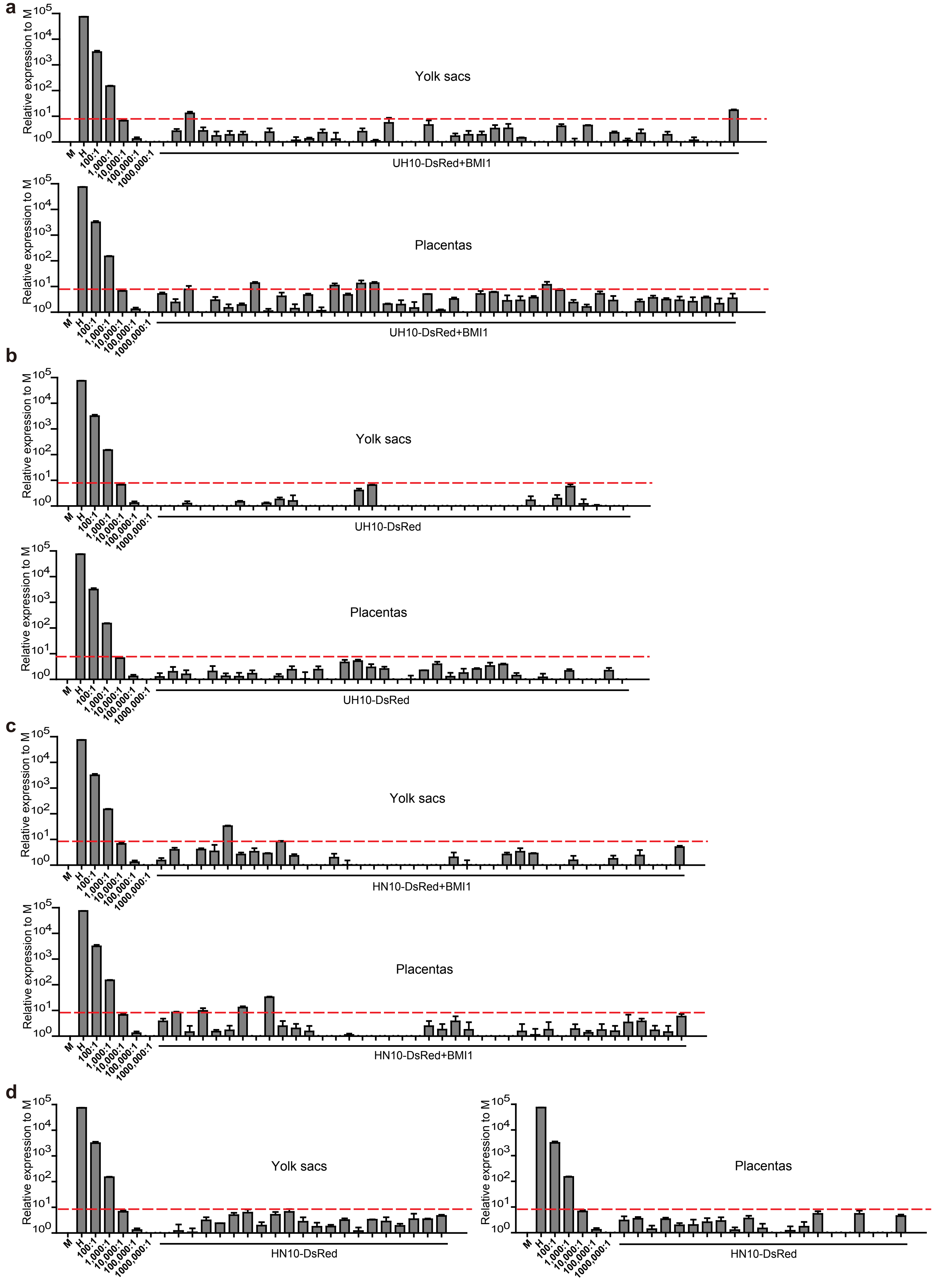

Supplementary Figure 4 hPSCs with BMI1 expression contribute to extra-embryonic tissues in mouse chimera. (a-d) Quantitative genomic PCR analysis of the human mitochondria DNA in all recovered E10.5 mouse yolk sacs and placentas after injection of the indicated cells.

Supplementary Figure 5

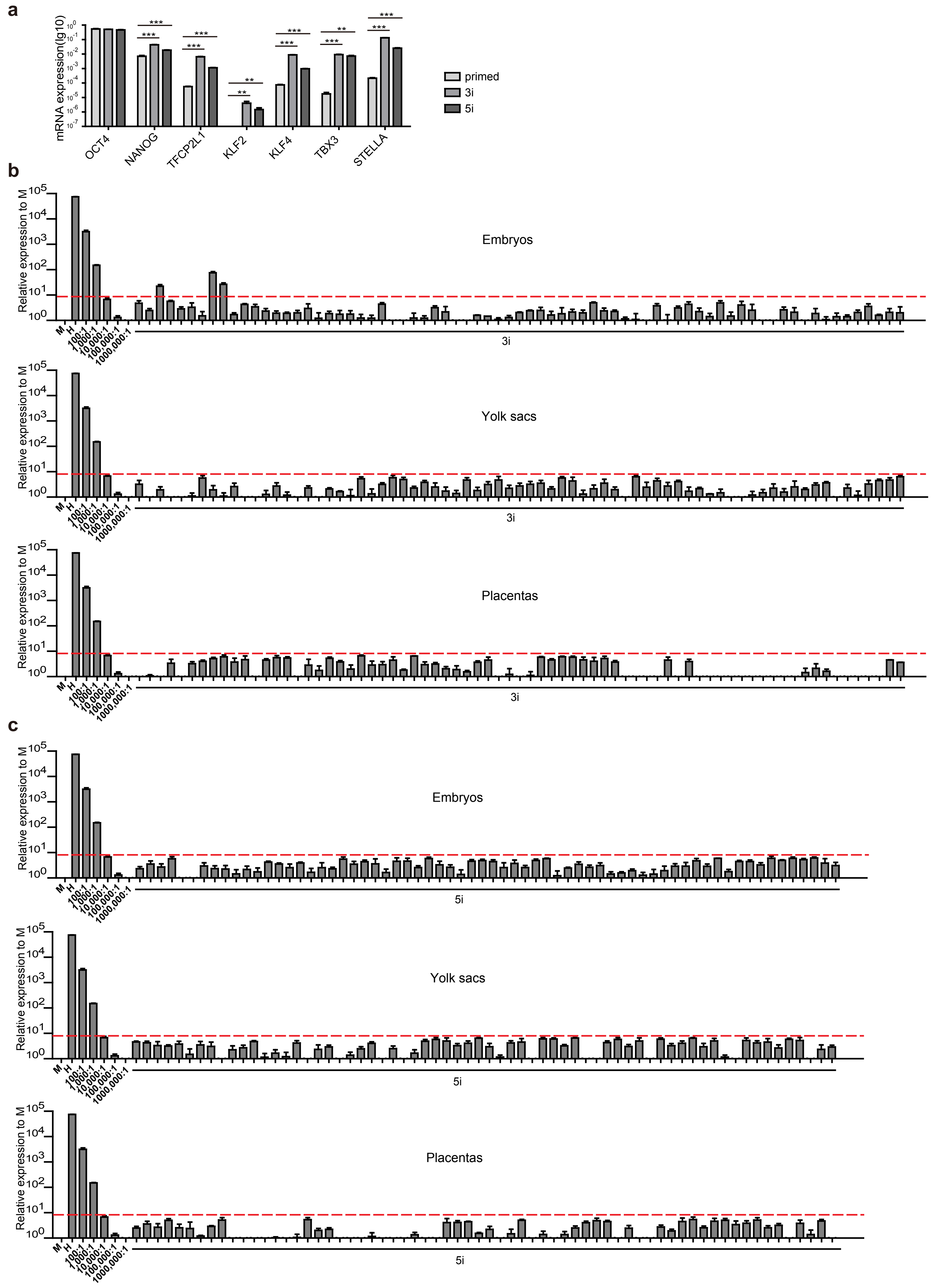

Supplementary Figure 5 Apoptosis is a major barrier in interspecies chimerism with niave hPSCs. (a) RT-qPCR analysis of the selected naïve state pluripotency markers in the indicated cells. Error bars represent mean + SEM of three independent replicates; **$p < 0.01$, ***$p < 0.001$. **(b-c)** Quantitative genomic PCR analysis of the human mitochondria DNA in all recovered E10.5 mouse embryos, yolk sacs and placentas after injection of the 3i and 5i cells, respectively.

Supplementary Table 1 Primers

NANOG-R	CCAGTGTCCAGACTGAAATTGAGT	RT-qPCR
KLF4-F	CAGCTTCACCTATCCGATCCG	RT-qPCR
KLF4-R	GACTCCCTGCCATAGAGGAGG	RT-qPCR
TFCP2L1-F	CAGCCCGAGCACTACAACC	RT-qPCR
TFCP2L1-R	CTCCCAGCTTCCGATTCTCC	RT-qPCR
OCT4-F	GCTCGAGAAGGATGTGGTCC	RT-qPCR
OCT4-R	CGTTGTGCATAGTCGCTGCT	RT-qPCR
REX1-F	GGAATGTGGGAAAGCGTTCGT	RT-qPCR
REX1-R	CCGTGTGGATGCGCACGT	RT-qPCR
STELLA-F	GTTACTGGGCGGAGTTCGTA	RT-qPCR
STELLA-R	TGAAGTGGCTTGGTGTCTTG	RT-qPCR
GBX2-F	CCGCCTTCAGCATAGACTCG	RT-qPCR
GBX2-R	GGTAGCCGGTGTAGACGAAAT	RT-qPCR
SALL4-F	AGCACATCAACTCGGAGGAG	RT-qPCR
SALL4-R	CATTCCCTGGGTGGTTCACTG	RT-qPCR
TBX3-F	GAGGCTAAAGAACTTTGGGATCA	RT-qPCR
TBX3-R	CATTTCGGGGTCGGCCTTA	RT-qPCR
hmtDNA-F	AATATTAAACACAAACTACCACCTACCT	RT-qPCR
hmtDNA-R	TGGTTCTCAGGGTTTGTTATAA	RT-qPCR
UCNE-F	AACAATGGGTTCAGCTGCTT	RT-qPCR
UCNE-R	CCCAGGCGTATTTTTGTTCT	RT-qPCR

Supplementary Table 2 Antibodies

Antibody	Source	Cat.no	Dilution Factor
Rabbit Anti-Human BMI1	Cell Signaling Techenology	5856	1：1000
DAPI	Sigma	D9542	1：2000
Mouse Anti-Human Stem121	Cellartic	Y40410	1：500
Rabbit Anti-Human Calponin	Abcam	AB46794	1：500
Rabbit Anti-Human PAX6	Biolegend	901301	1：300
Mouse Anti-Human SOX17	R&D System	MAB1924	1：200
Mouse Anti-Human OCT4	Santa Cruz Biotechnology	sc-5279	1：200
Rabbit Anti-Human CDX2	Cell Signaling Techenology	3977	1：200
Mouse Anti-Human CK7	Zsbio	ZM-0071	1：200
Rabbit Anti-TPBPA (Trophoblast specific protein alpha)	Abcam	ab104401	1：1000
HRP-conjugated Monoclonal Mouse Anti-GAPDH	KangChen	KC-5G5	1：1000
Goat Anti-Rabbit IgG HRP	KangChen Bio-tech	KC-RB-035	1：4000
Goat Anti-Mouse IgG (Alexa Fluor 488)	Abcam	ab150113	1：500
Goat Anti-Rabbit IgG (Alexa Fluor 488)	Thermo Fisher SCIENTIFIC	A-11008	1：500
Goat Anti-Mouse IgG (Alexa Fluor 647)	Abcam	ab150115	1：500
